# Trailblazer in Health Education: The Legacy of Thomas Denison Wood

**DOI:** 10.7759/cureus.70134

**Published:** 2024-09-24

**Authors:** Anita M, Bhuminathan S, Charumathi Dhanushkodi, Suganya P, Mohan Valiathan

**Affiliations:** 1 Dental Sciences, Bharath Institute of Higher Education and Research, Chennai, IND; 2 Public Health Dentistry, Sree Balaji Dental College and Hospital, Chennai, IND; 3 Prosthodontics and Crown and Bridge, Sree Balaji Dental College and Hospital, Bharath Institute of Higher Education and Research, Chennai, IND; 4 Public Health Dentistry, Sree Balaji Dental College and Hospital, Bharath Institute of Higher Education and Research, Chennai, IND; 5 Periodontics and Implantology, Sree Balaji Dental College and Hospital, Bharath Institute of Higher Education and Research, Chennai, IND

**Keywords:** health education, health examination, historical vignette, physical education, school sanitation

## Abstract

Thomas Denison Wood, often regarded as the "Father of Health Education," was a pioneering figure in the early 20th century whose work laid the foundation for modern health education. His contributions were instrumental in integrating health education into the public school curriculum in the United States. Wood advocated for a holistic approach to health, encompassing physical fitness, hygiene, nutrition, mental health, and preventive care. His key achievements include the development of comprehensive health education programs, leadership in professional organizations, and the establishment of the New York City School Health Program. Wood authored several influential books, such as "Health by Stunts" (1901) and "A Textbook of Hygiene" (1915), which provided practical guidance on maintaining health and were widely used in educational settings. His work emphasized the importance of lifelong health education, beginning in childhood and continuing throughout life. Through his writings, teaching, and advocacy, Wood significantly shaped the field of health education, and his legacy continues to influence health education practices today.

## Introduction and background

Traditionally, the ideology of schools was to strictly focus on academics rather than on the health and wellness of the child. However, the innovative thoughts of Thomas Dennison Wood were more scientifically structured. They had a greater impact on both the students and community health and paved the way for the implementation of various programs in the United States: the Move-to-Improve, Whole School, Whole Community, Whole Child (WSCC) model, and Comprehensive School Physical Activity Programs. A comprehensive search of databases including Cochrane Library, Wiley Online Library, Science Direct, and PubMed was conducted. Keywords used were “health education” AND “child health” AND “hygiene.”

Wood, born in 1865, is a central figure in the history of health education in the United States. Often called the "Father of Health Education," Wood's work was instrumental in establishing health education as a vital component of the public school curriculum, laying the groundwork for the field as it exists today [1]. His innovative ideas about integrating health into education have impacted both public health and educational systems worldwide.

**Figure 1:**
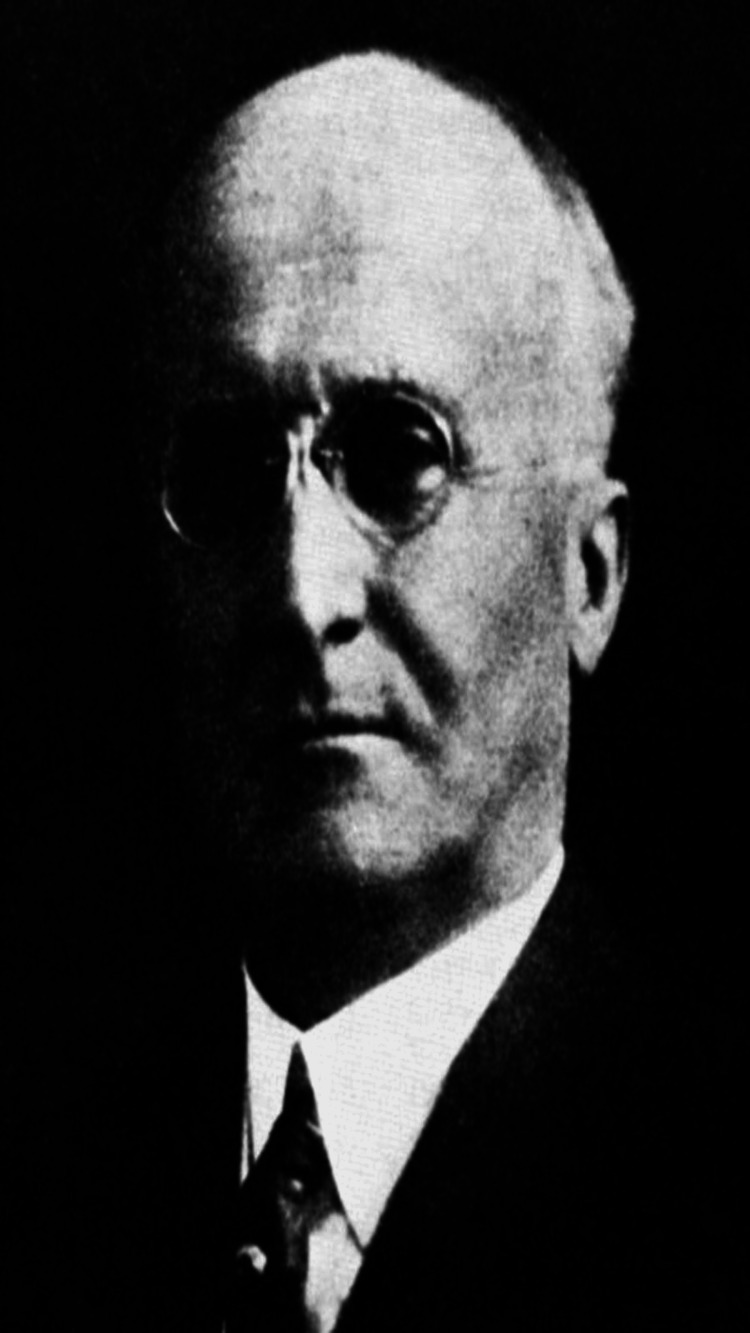
Photograph of Thomas Dennison Wood Image Credit: Davenport, 1984 [2]; published with permission. Photo courtesy of Teachers College, Columbia University, New York

Wood was born in Sycamore, Illinois, in the United States in 1865, during which significant societal changes were brought on by industrialization and urbanization. These developments presented new public health challenges, such as the spreading of infectious diseases and unhealthy living conditions in crowded urban areas. Growing up in this environment, Wood became keenly aware of the connection between health and education, eventually recognizing that education could be a powerful tool in improving public health outcomes [2]. Wood, after completing his physical education graduation from Oberlin in 1889, earned his A.M. and M.D. degrees from Columbia University. Figure 1 shows his photograph obtained from Columbia University, New York.

He pursued his education at Bowdoin College, followed by a medical degree from the University of Pennsylvania in 1890. This unique combination of training in both medicine and education gave Wood a broad perspective, enabling him to bridge the gap between these two fields [3]. His interdisciplinary approach became a defining characteristic of his work in health education.

## Review

**Figure 2:**
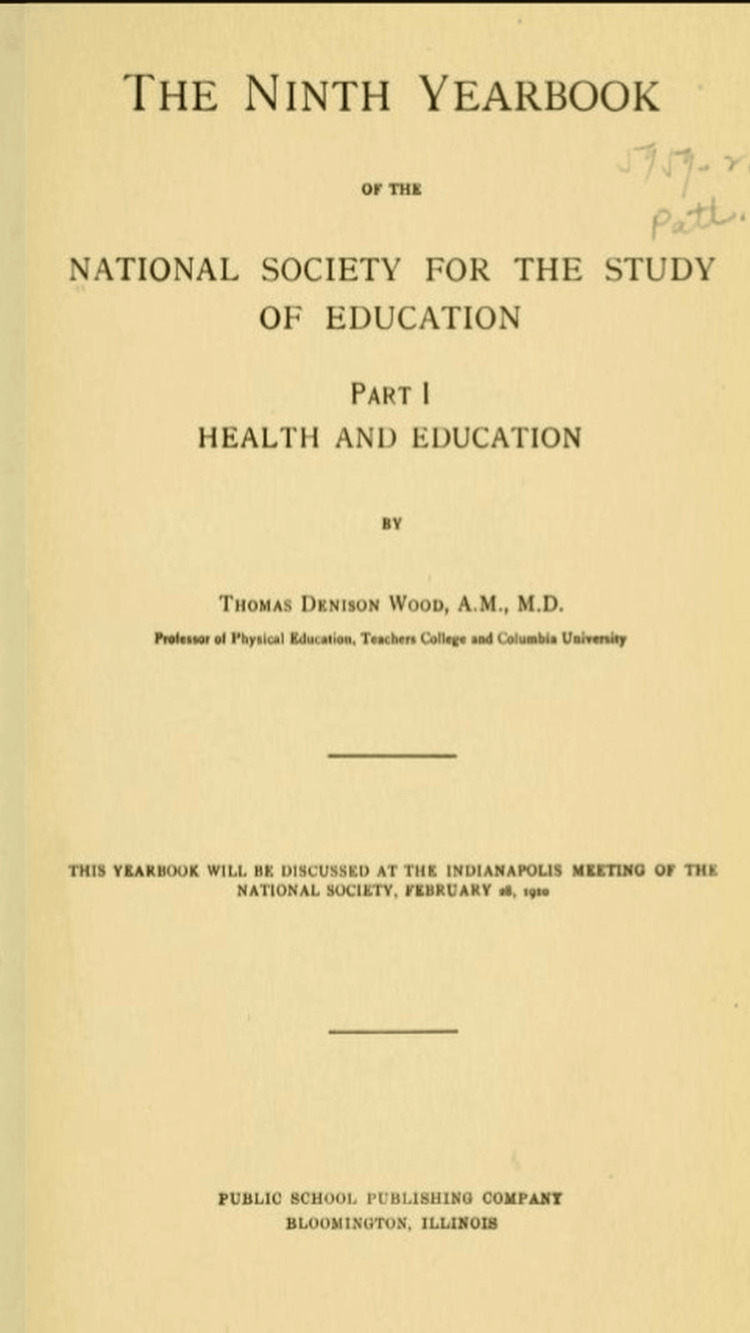
Book by Thomas Denison Wood published as the ninth yearbook: Health and Education [1]

In the early 20th century, health education was not yet a formalized field, and education focused mainly on academic subjects. Health education was often perceived as a responsibility of medical professionals like doctors and nurses rather than as a social responsibility, and it often lacked a curriculum. However, Wood envisioned a more comprehensive role for education, where schools would not only impart knowledge but also promote the health and well-being of students and the community in a framework that included lessons on nutrition, personal hygiene, physical fitness, and emotional well-being. He advocated for this holistic approach to health education that addressed physical, mental, and emotional well-being, arguing that schools should foster healthy lifestyles and habits that students would carry into adulthood. One of his greatest accomplishments is his book on “Health and Education,” which was published every year consequently for more than 10 years (Figure 2).

Wood's vision extended beyond basic hygiene instruction. He believed in a comprehensive curriculum that included physical education, nutrition, mental health, and preventive care. This approach was revolutionary at the time and set the stage for developing health education as a distinct field. He introduced sports and games to the classes for which student interests were high from the beginning and also established degree programs on physical education and summer school for teachers.

Key contributions

Development of Comprehensive Health Education Programs

One of Wood's most significant contributions was his role in developing comprehensive health education programs within the public school system. At a time when education was largely focused on academic achievement, Wood emphasized the need for schools to play a central role in promoting health. He championed a curriculum that included physical education, hygiene, nutrition, and mental health, aiming to produce well-rounded and healthy individuals. His holistic approach was innovative and laid the groundwork for future developments in health education [4].

Advocacy for School Health Programs

Wood was a key figure in establishing some of the earliest school health programs in the United States. His work in developing the New York City School Health Program was particularly notable. This program, launched in the early 1900s, was one of the first of its kind and served as a model for other school districts across the country. It included regular health examinations for students, hygiene education, and the promotion of physical activity, all of which were groundbreaking at the time [5]. Wood's involvement in this initiative helped to institutionalize health education within the public school system, setting a precedent for future programs.

Leadership in Professional Organizations

Beyond his direct contributions to education, Wood was also a prominent leader in various professional organizations, including the American Public Health Association and the American Physical Education Association. Through these platforms, he advocated for the recognition of health education as an essential field. His leadership within these organizations helped to shape policies and practices that advanced health education on both state and national levels [6]. Wood’s influence extended beyond the classroom, impacting the broader landscape of public health and education.

Integration of Physical Education and Health

Wood believed that physical education was a critical component of overall health education. He argued that physical fitness was essential not only for physical health but also for mental well-being. At Columbia University, where he served as a professor and later as the head of the Department of Physical Education and Health, Wood worked to integrate physical education into the broader health education curriculum [7]. He developed programs that combined physical exercise with lessons on nutrition, hygiene, and disease prevention, anticipating modern concepts of holistic health education.

**Figure 3:**
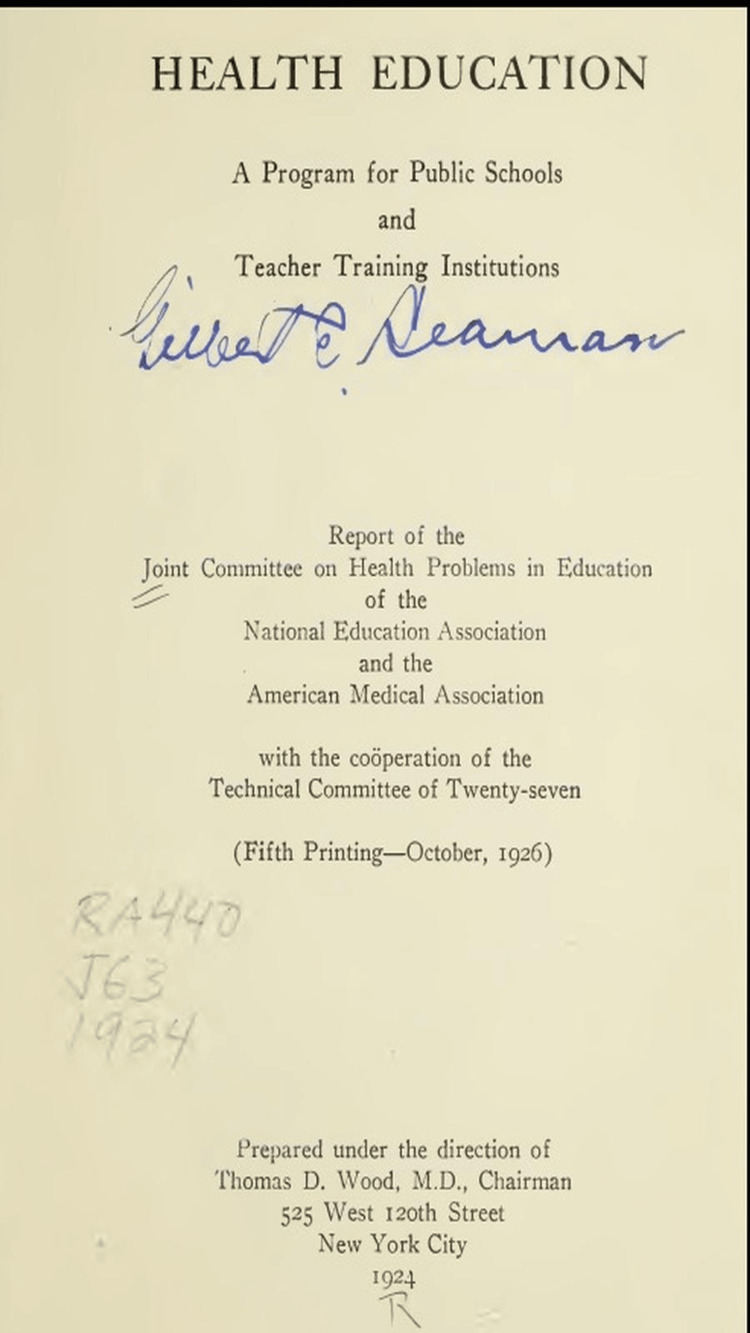
Book by Thomas Denison Wood in 1924: Health Education – A Program for Public Schools and Training Institutions [7]

Wood's contributions to health education

Wood was a prolific writer, and his published works played a significant role in disseminating his ideas about health education. Some of his most influential books include the following:

Health by Stunts (1901)

Co-authored with Robert Tait McKenzie, "Health by Stunts" was one of the first books to emphasize the importance of physical activity for maintaining health. The book provided practical exercises that could be performed by students and adults alike, promoting the idea that regular physical activity was crucial for a healthy life. The term “stunts” referred to simple exercises that required no specialized equipment, making them accessible to a wide audience [8]. This book was influential in popularizing the concept of physical fitness as a component of health education.

The Child in the School (1907)

In "The Child in the School," Wood highlighted the importance of addressing children's health needs within the school environment. He argued that schools had a responsibility to promote the physical and mental well-being of their students. The book covered a wide range of topics, including the importance of regular health inspections, the role of teachers in health education, and the need for hygienic school facilities [9]. "The Child in the School" was influential in shaping policies related to school health services and underscored the importance of a holistic approach to child health.

A Textbook of Hygiene (1915)

This textbook was aimed at both students and educators, providing a comprehensive overview of hygiene practices and their importance in maintaining health. Topics covered included personal cleanliness, sanitation, disease prevention, and nutrition. The book was widely used in schools and became a standard reference for health education during the early 20th century [10]. Its practical approach made it an essential resource for teachers and students alike.

Health Education in Elementary Schools (1927)

In this book, Wood outlines a detailed curriculum for health education in elementary schools. It provided lesson plans, activities, and educational materials that teachers could use to promote health among young students. Wood emphasized the importance of starting health education early in a child's life, with a focus on establishing healthy habits that would last into adulthood. This book helped to standardize health education practices in schools across the country.

In this book, he has also quoted about oral hygiene, the importance of nutrition in health, eye hygiene, and social, mental, and physical health. He has even mentioned the influence of adverse habits like smoking, alcohol, and drugs on the general health of school-going adolescents [1].

Health and Education (1932)

**Figure 4:**
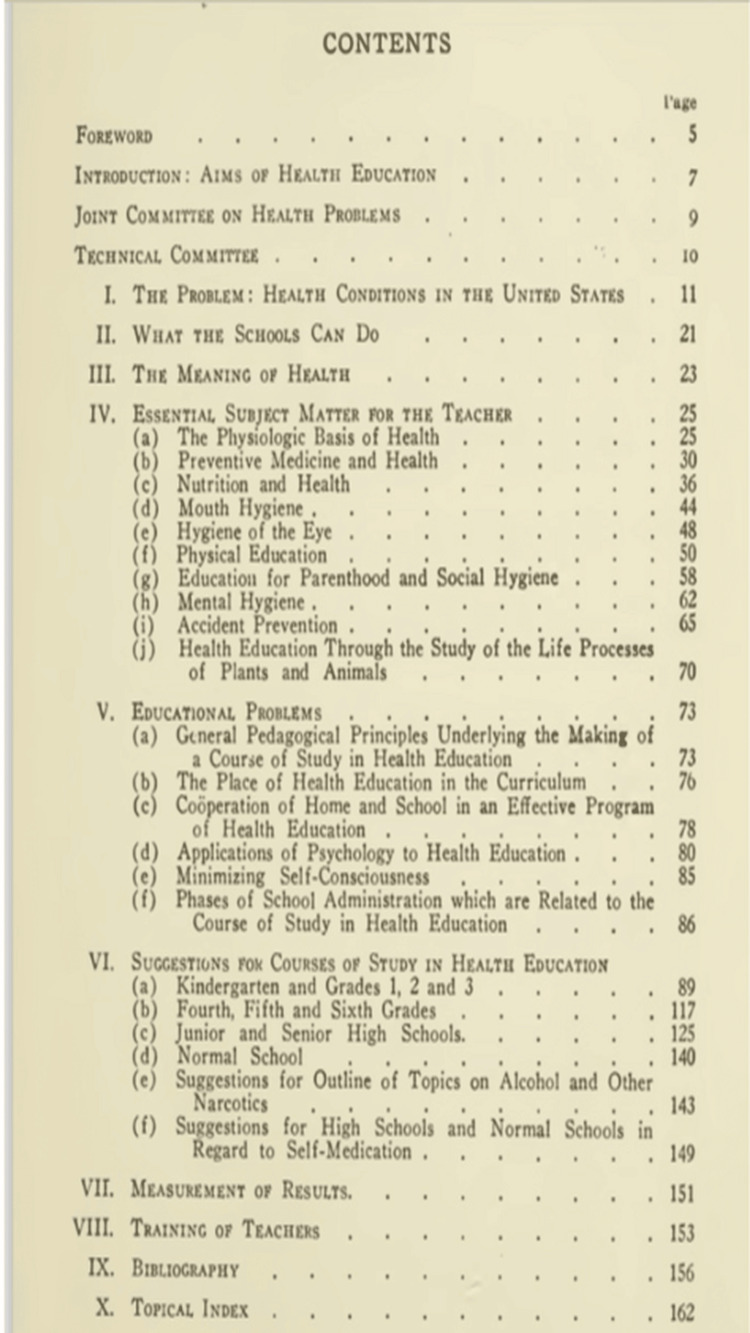
Contents of the book Health and Education by Thomas Denison Wood explaining the health condition of the United States at that time, the meaning of health, health education material for teachers and students, and educational problems faced during the course of health education [1]

Co-authored with other experts in the field, this book was one of the first to address both health education and physical education as interconnected disciplines. It provided a detailed curriculum for integrating these two areas within schools, with chapters on physical fitness, mental health, nutrition, and hygiene. "Health and Physical Education" was influential in establishing the idea that health education should be a lifelong process, starting in childhood and continuing throughout one’s life [1]. The contents of this book are depicted in Figure 4. This work further solidified Wood's reputation as a leading figure in health education.

Moreover, Wood's influence extended beyond education. His work raised awareness about the importance of public health and the role that education could play in promoting it. This contributed to the development of public health policies and programs aimed at improving health outcomes on a broader scale. Wood’s advocacy for integrating health education into the school curriculum was a significant step toward institutionalizing public health practices in educational settings [11].

Legacy and continued relevance

The principles he advocated for holistic, preventive, and lifelong health education are now widely accepted and implemented in schools around the world. Health education is considered essential to the development of healthy, informed citizens, and Wood's vision has played a critical role in shaping this perspective. The ongoing relevance of Wood's work is evident in the continued efforts to address public health challenges through education. In an era where issues such as obesity, mental health, and chronic diseases are of growing concern, the need for comprehensive health education is more critical than ever [5]. Wood's vision of an education system that promotes not just academic achievement but also the overall well-being of students remains as pertinent today as it was over a century ago.

In recognition of his contributions, Wood has been honored by various organizations and institutions. His work continues to be studied and celebrated by educators, public health professionals, and historians alike [12].

## Conclusions

Wood was a visionary whose work laid the foundation for the field of health education. His efforts to integrate health education into the school curriculum and his advocacy for a holistic approach to health have had a lasting impact on both education and public health. "Father of Health Education," Wood's legacy continues to influence the way we think about and approach health education today. Through his writings, teaching, and advocacy, Wood significantly shaped the field of health education. His work serves as a reminder of the enduring importance of education in promoting public health and well-being. The principles he championed over a century ago continue to inform and inspire the practice of health education today, highlighting the lasting impact of his contributions on the health and well-being of society.
